# The associations between speed performance and health outcomes in children and adolescents: A systematic review and meta-analysis

**DOI:** 10.1016/j.jesf.2026.200461

**Published:** 2026-02-26

**Authors:** Yuhang Yang, Jin Wu, Jing He, Jiuzhang Li, Jane Jie Yu

**Affiliations:** a Department of Sports Science, College of Education, Zhejiang University, No.866, Yuhangtang Road, Xihu District, Hangzhou, China; b Department of Physical Education, Zhejiang Wanli University, No.8, Qianhu South Road, Yinzhou District, Ningbo, China; c College of Physical Education, Shanghai Normal University, No.100, Haixi Road, Fengxian District, Shanghai, China; d Discipline of Exercise & Sport Science, Faculty of Medicine & Health, The University of Sydney, Magglingen, NSW, 2006, Australia

**Keywords:** Running, Physical fitness, Adiposity, Bone development, Cardiometabolic risk factors

## Abstract

**Objectives:**

To synthesize current evidence on the associations between speed performance and health outcomes in children and adolescents.

**Methods:**

A systematic search of four databases was conducted up to March 31, 2025. Studies quantifying associations between speed performance and health parameters in generally healthy individuals aged 3-19 years were included. Pooled correlation coefficients and 95% CI were derived from the *r* values and sample sizes of included studies.

**Results:**

A total of 58 studies involving 481,579 individuals (232,666 girls) were included. Meta-analysis results indicate significant (all *p* < 0.05) low-to-moderate associations of speed performance with body mass index (*r* = 0.179), fat-free mass (*r* = −0.318), fat mass (*r* = 0.247), percentage of body fat (*r* = 0.352), sum of skinfolds (*r* = 0.271), waist circumference (*r* = 0.211), triglycerides (*r* = 0.191), bone mineral content (*r* = −0.382), bone mineral density (*r* = −0.398), bone speed of sound (*r* = −0.256), anxiety (*r* = 0.258), and physical self-concept (*r* = −0.436). These associations were moderated by relevant variables, with moderating patterns differing across specific health outcomes. Evidence regarding the associations of speed performance with blood pressure and depression was inconclusive.

**Conclusion:**

This study revealed significant associations between speed performance and various health outcomes in children and adolescents, suggesting that speed performance assessment could be useful for the early identification of those at health risk within this population.

## 1 Introduction

Physical fitness, recognized as a powerful health marker,^1^ is commonly classified into two principal components known as health-related physical fitness (HRF) and skill-related physical fitness (SRF).^2^ HRF refers to the ability to perform daily activities with sufficient energy and the traits associated with reducing the risk of chronic diseases,^3^ and it traditionally encompasses cardiorespiratory fitness, muscular fitness, body composition, and flexibility.^2^ SRF, on the other hand, includes speed, power, coordination, reaction time, agility, and balance, which were traditionally viewed as being primarily oriented toward sport performance and motor skills.^4^

However, with advancing research, this conceptual classification has been increasingly reconsidered, particularly in pediatric populations, whose daily physical activity is typically spontaneous, intermittent, and dominated by short bouts of high-intensity effort that rely heavily on anaerobic energy pathways.^5^ From a developmental and physiological perspective, associated fitness components, most notably speed and power, are therefore considered relevant to growth and maturation processes, likely through mechanisms involving neuromuscular development and anabolic regulation.^5^

Reflecting this evolving perspective, several widely used standardized fitness batteries for children and adolescents, such as the ALPHA^6^ and PREFIT,^7^ have incorporated speed performance within their HRF domains. However, the empirical foundation for this inclusion remains incomplete. Since HRF is defined primarily by its relevance to health, establishing the strength and breadth of the relationship between speed performance and health represents a necessary initial step. While determining its unique contribution is a crucial subsequent goal, the primary task of systematically synthesizing the existing evidence has not yet been performed. Indeed, the specific associations between speed performance and health have received relatively scant attention in the published literature, particularly when compared to the extensive evidence available for cardiorespiratory fitness^8^, ^9^, ^10^ and muscular fitness,^11^, ^12^, ^13^ leaving the potential of speed performance as a health marker insufficiently quantified. Specifically, although existing systematic reviews suggest that speed performance, rather than cardiorespiratory fitness, has a positive effect on skeletal health,^1^^,^^14^ and evidence also associates speed performance with overweight and obesity,^15^ quantitative syntheses remain scarce. Few systematic reviews with meta-analysis have synthesized the available evidence, and these have been primarily limited to the psychological health domain.^16^ To our knowledge, there is currently no systematic review providing a quantitative synthesis covering a broad range of health outcomes. Therefore, the purpose of this study is to conduct a systematic review and meta-analysis of the evidence regarding the association of speed performance with health outcomes in children and adolescents.

## 2 Methods

### 2.1 Protocol and registration

This study adhered to the PRISMA statement, with a prospectively registered protocol in PROSPERO (reference number: CRD42023444668).^17^

### 2.2 Data sources and search

The databases of Web of Science, Scopus, PubMed and China National Knowledge Internet were searched online from inception to March 31, 2025. The search strategy was built around three conceptual categories of keywords: population, speed performance, and health outcome. The full search strategy is available in Supplemental Material.

### 2.3 Eligibility criteria

Inclusion criteria has been defined as follows: (i) study participants were generally healthy individuals aged 3 to 19 years; (ii) the study presented a quantitative evaluation of speed performance, operationally defined as the ability to move the body rapidly, encompassing both linear sprint tests (e.g., 50 m sprint) and speed shuttle run tests that rely primarily on the anaerobic energy system (e.g., 4 × 10 m speed shuttle run)^18^^,^^19^; (iii) study quantified the association between speed performance and health parameters; (iv) data obtained from cross-sectional studies, longitudinal studies, and experimental studies; (v) English or Chinese or Spanish-language peer-reviewed journal articles about this subject. Exclusion criteria have been defined as follows: (i) special participants (e.g., athletes or people with chronic or acute illnesses or injuries); (ii) studies for which correlation coefficients were neither reported nor available.

### 2.4 Study selection and data extraction

**Fig. 1:**
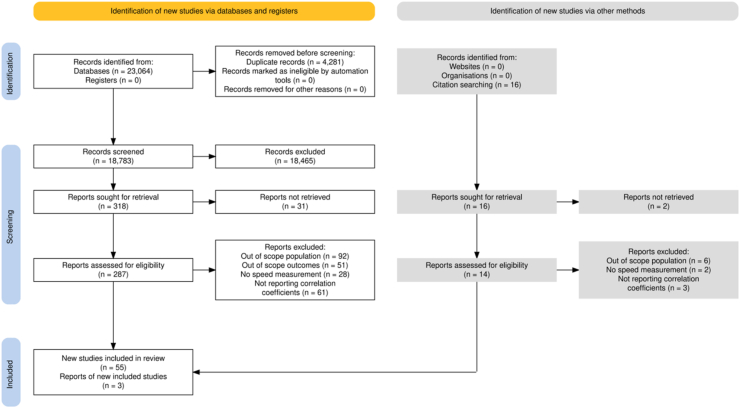
Flowchart of study selection.

The literature screening process consisted of two stages. In the first stage, the titles and abstracts of the search results were reviewed. In the second stage, those studies that appeared to meet the criteria were read in full text. The inter-rater reliability between the two reviewers across both stages was assessed using the κ statistic. The selection process was conducted by two independent authors (YY and JW). When the inclusion of a study was unclear, a third author (JH) was consulted, and any disputes were resolved by discussion among these authors. The PRISMA flowchart is shown in Fig. 1.^20^

### 2.5 Risk of bias assessment

Risk of bias (RoB) was independently assessed by two authors (YY and JW), and any discrepancies were settled in a consensus meeting. Based on the results of previous studies, we used the Joanna Briggs Institute Critical Appraisal Checklist as the RoB assessment tool.^21^ The bias risk classification for each study was based on the proportion of checklist items that met the “yes” criteria: low risk if > 70%, moderate risk if 50–69%, and high risk if < 50%.

### 2.6 Data synthesis and meta-analysis

Meta-analyses were performed only when a minimum of three studies reported effect sizes for the same health parameter.^22^ Consequently, due to the scarcity of longitudinal data (see Results), quantitative synthesis was restricted to cross-sectional associations (including baseline data from longitudinal studies). This meta-analysis was based on correlation (*r*) coefficient. Studies employing regression analysis were excluded if correlation coefficients were unreported and unavailable from the authors.^23^

Based on existing studies,^24^, ^25^, ^26^ both zero-order and partial correlation coefficients (e.g., age and sex) were extracted from each study and entered into the statistical program along with its sample size. In order to obtain approximately normally distributed values, each correlation coefficient was transformed into a Fisher's z-score and standard error.^27^^,^^28^ We used Cohen's cut-offs of 0.10, 0.30, and 0.50 to account for the correlation's strength. 0.10 ≤ *r* < 0.30 as small correlated, 0.30 ≤ *r* < 0.50 as medium correlated, and *r* ≥ 0.50 as large correlated.^29^

Heterogeneity was assessed using the Q-test and *I*^2^ statistic. Given the inherent clinical and methodological heterogeneity across the included studies, a random-effects model was employed for all meta-analyses.^28^ Publication bias was analyzed using the Classic Fail-safe N^30^ and Egger's test.^31^ The absence of significant publication bias was indicated when the Fail-safe N exceeded the critical threshold of 5*k* + 10 (where *k* represents the number of effect sizes), or when Egger's test yielded a statistically significant result (*p* < 0.05). Should publication bias be detected, the Trim-and-Fill method was performed for correction.^32^ Sensitivity analyses were carried out by removing each study from the model once to identify the potential causes of heterogeneity and assess the stability of the meta-analysis results. To examine sources of heterogeneity and the moderating effects of different factors on the association between speed performance and various health parameters, this study conducted subgroup analysis for categorized moderators containing at least two effect sizes and meta-regression for continuous moderators including at least twenty effect sizes.^33^^,^^34^ The Comprehensive Meta-Analysis program (version 3; Biostat, Englewood, NJ, USA) was used for all statistical analyses. Pooled effect sizes were estimated using the inverse-variance weighted random-effects model. The between-study variance (τ^2^) was calculated using the DerSimonian-Laird estimator. No specific small-sample adjustments (e.g., Hartung-Knapp) were applied to the standard errors. *p* < 0.05 was chosen as the threshold for significance in this meta-analysis.

### 2.7 Certainty of evidence assessment

The ‘Grading of Recommendations Assessment, Development and Evaluation’ (GRADE) instrument was utilized to appraise the certainty of evidence for each primary outcome.^35^^,^^36^ Two authors (YY and JY) independently assessed the certainty of evidence for each primary outcome, with any discrepancies resolved through discussion.

### 2.8 Deviations from the registered protocol

The registered protocol for this review initially planned for the quantitative synthesis of longitudinal associations. However, during data extraction, it became evident that fewer than three studies reported longitudinal associations for any single health outcome. Given the insufficient data to support a robust longitudinal meta-analysis, the quantitative synthesis was restricted to cross-sectional associations (including baseline data from longitudinal studies). This deviation is transparently described here in accordance with PRISMA 2020 guidelines to ensure the reliability and clarity of the findings.

## 3 Results

### 3.1 Literature search and selection

Following initial database searching and supplementary citation searching, potential studies were screened by title and abstract. After full-text review against the eligibility criteria, 58 articles were deemed eligible and included in the meta-analysis. Inter-rater reliability was excellent for both title and abstract screening (κ = 0.79) and full-text screening (κ = 0.88).

### 3.2 Study characteristics

Table S2 presents the characteristics of the 58 studies included in this meta-analysis.

These studies included 481,579 individuals (232,666 girls), having sample sizes that range from 69^37^ to 177,419.^38^ Participants included only girls (n = 2),^39^^,^^40^ boys (n = 5),^41^, ^42^, ^43^, ^44^, ^45^ or both (n = 51)^35^, ^36^, ^46^, ^47^, ^48^, ^49^, ^50^, ^51^, ^52^, ^53^, ^54^, ^55^, ^56^, ^57^, ^58^, ^59^, ^60^, ^61^, ^62^, ^63^, ^64^, ^65^, ^66^, ^67^, ^68^, ^69^, ^70^, ^71^, ^72^, ^73^, ^74^, ^75^, ^76^, ^77^, ^78^, ^79^, ^80^, ^81^, ^82^, ^83^, ^84^, ^85^, ^86^, ^87^, ^88^, ^89^, ^90^, ^91^, ^92^, ^93^, ^94^.

Among the included studies, the majority utilized a cross-sectional design (n = 56),^37^, ^38^, ^39^, ^40^, ^41^, ^42^, ^43^, ^44^, ^45^, ^46^, ^47^, ^48^^,^ 50, 51, 52, 53, 54, 55, 56, 57, 58, 59, 60, 61, 62, 63, 64, 65, 66, 67, 68, 69, 70, 71, 72, 73, 74, 75, 76, 77, 78, 79, 80, 81, 82, 83, 84, 85, 86, 87, 88, 89, 90, 91, 92, 93^,^ ^37^ alongside one quasi-experimental study^49^ and one longitudinal study.^94^ It should be noted that our systematic search identified fewer than three studies reporting longitudinal associations for any single health parameter. Consequently, given the insufficiency of data for a longitudinal meta-analysis, we extracted only the baseline measurements from one longitudinal study^94^ and incorporated them into the cross-sectional synthesis.

Based on the geographic classifications defined by the World Health Organization, the included studies were distributed across the following five regions: the African Region (AFRO, n = 1),^44^ the Region of the Americas (AMRO, n = 7),^47^^,^^63^^,^^64^^,^^70^^,^^74^^,^^75^^,^^79^ the European Region (EURO, n = 36),^37^, ^39^, ^40^, ^41^^,^^45^^,^^48^, ^49^, ^50^, ^51^, ^52^, ^53^^,^^55^^,^^57^^,^^59^, ^60^, ^61^, ^62^^,^^65^, ^66^, ^67^^,^^69^^,^^71^, ^72^, ^73^^,^^76^, ^77^, ^78^^,^^82^, ^83^, ^84^, ^85^, ^86^^,^^88^, ^89^, ^90^^,^^94^ the Eastern Mediterranean Region (EMRO, n = 3),^42^^,^^43^^,^^46^ and the Western Pacific Region (WPRO, n = 11).^38^^,^^54^^,^^56^^,^^68^^,^^80^^,^^81^^,^^87^^,^^91^, ^92^, ^93^.

The study subjects ranged in age from 3 to 19 years. Four studies involved only preschoolers (age ≤6),^46^^,^^59^^,^^73^^,^^92^ 52 studies involved only school-age children and/or adolescents (6 < age ≤19),^38^, ^39^, ^40^, ^41^, ^42^, ^43^, ^44^, ^45^^,^ 47, 48, 49, 50, 51, 52, 53, 54, 55, 56, 57, 58^,^ 60, 61, 62, 63, 64, 65, 66, 67, 68, 69, 70, 71^,^ 74, 75, 76, 77, 78, 79, 80, 81, 82, 83, 84^,^ 86, 87, 88, 89, 90, 91^,^ 37, 93, 94 two study involved both preschoolers and school-age children.^72^^,^^85^

The sprint (n = 36)^38^^,^^41^, ^42^, ^43^, ^44^, ^45^, ^46^^,^^51^^,^^52^^,^^54^, ^55^, ^56^, ^57^, ^58^^,^^61^, ^62^, ^63^^,^^65^, ^66^, ^67^, ^68^^,^^72^^,^^74^^,^^75^^,^^78^, ^79^, ^80^, ^81^^,^^84^^,^^86^, ^87^, ^88^, ^89^^,^^91^^,^^37^, ^93^, ^94^ and the speed shuttle run (n = 22)^37^, ^39^^,^^40^^,^^47^, ^48^, ^49^, ^50^^,^^53^^,^^59^^,^^60^^,^^64^^,^^69^^,^^71^^,^^73^^,^^76^^,^^77^^,^^82^^,^^83^^,^^85^^,^^90^^,^^92^ were the most commonly used test types to assess speed performance.

The health outcomes of studies can primarily be categorized into the following aspects: anthropometric and adiposity parameters (n = 32),^38^, ^39^, ^40^, ^41^^,^^43^, ^44^, ^45^, ^46^^,^^48^, ^49^, ^50^^,^^52^^,^^54^, ^55^, ^56^^,^^58^^,^^60^^,^^63^, ^64^, ^65^, ^66^, ^67^, ^68^, ^69^, ^70^, ^71^^,^^73^^,^^76^, ^77^, ^78^, ^79^, ^80^, ^81^^,^^85^, ^86^, ^87^, ^88^, ^89^^,^^91^, ^92^, ^93^, ^94^ cardiometabolic parameters (n = 4),^47^^,^^48^^,^^60^^,^^94^ bone parameters (n = 7),^59^^,^^61^^,^^62^^,^^72^^,^^74^^,^^75^^,^^90^ and psychological parameters (n = 8).^42^^,^^51^^,^^53^^,^^57^^,^^82^, ^83^, ^84^^,^^37^ Anthropometric and adiposity parameters mainly included body mass index (BMI, n = 30),^38^^,^^44^^,^^46^^,^^48^, ^49^, ^50^^,^^52^^,^^55^^,^^56^^,^^58^^,^^60^^,^^64^^,^^65^^,^^67^^,^^69^, ^70^, ^71^^,^^73^^,^^76^^,^^78^, ^79^, ^80^^,^^85^, ^86^, ^87^, ^88^, ^89^^,^^91^, ^92^, ^93^ percentage of body fat (BF%, n = 11),^41^^,^^43^, ^44^, ^45^, ^46^^,^^48^^,^^60^^,^^71^^,^^76^^,^^79^^,^^81^ waist circumference (WC, n = 9),^46^^,^^48^^,^^58^^,^^66^^,^^70^^,^^71^^,^^73^^,^^77^^,^^94^ fat mass (FM, n = 6),^41^^,^^60^^,^^68^^,^^76^^,^^77^^,^^88^ fat-free mass (FFM, n = 5),^54^^,^^60^^,^^68^^,^^76^^,^^79^ and sum of skinfolds (SSF, n = 5).^39^^,^^40^^,^^63^^,^^64^^,^^77^ Cardiometabolic parameters mainly included diastolic blood pressure (DBP, n = 3),^48^^,^^60^^,^^94^ systolic blood pressure (SBP, n = 3),^48^^,^^60^^,^^94^ and triglycerides (n = 3).^47^^,^^60^^,^^94^ Bone mineral content (BMC, n = 3),^59^^,^^74^^,^^90^ bone mineral density (BMD, n = 3),^59^^,^^74^^,^^75^ and bone speed of sound (SoS, n = 3)^61^, ^62^, ^72^ were the most commonly used bone parameters. Psychological parameters mainly included anxiety (n = 4),^51^^,^^57^^,^^82^^,^^83^ depression (n = 4),^42^^,^^51^^,^^82^^,^^83^ and physical self-concept (n = 4).^37^, ^53^^,^^82^^,^^84^

### 3.3 Risk of bias assessment results

Of the 58 studies that our meta-analysis included, no study was categorized as ‘high RoB’. The percentages of ‘low RoB’ and ‘moderate RoB’ were 72.41% and 27.59%, respectively (Supplementary material, Table S3).

### 3.4 Heterogeneity analysis results

Table 1 presents the heterogeneity analysis results. Statistical heterogeneity was low for the associations between speed performance and both BMC and depression (*p* > 0.1, *I*^2^ < 50%), whereas substantial heterogeneity was observed for other health parameters (*p* < 0.1, *I*^2^ > 50%).

**Table 1:** Results of heterogeneity and publication bias analysis.

Outcome Variable	n	*k*	Heterogeneity				Publication bias	
			*QB*	*df*	*p*	*I*^2^	*Fail-safe N*	Egger's test (*p*)
Body mass index	30	59	2566.471	58	<0.001	97.740	65791	0.587
Fat-free mass	5	6	175.520	5	<0.001	97.151	1042	0.045
Fat mass	6	7	42.340	6	<0.001	85.829	318	0.159
Percentage of body fat	11	24	91.324	23	<0.001	74.815	6085	0.055
Sum of skinfolds	5	20	65.120	19	<0.001	70.823	3013	0.850
Waist circumference	9	12	61.228	11	<0.001	82.034	594	0.055
Diastolic blood pressure	3	3	6.010	2	0.050	66.722	2	0.085
Systolic blood pressure	3	3	5.270	2	0.072	62.052	0	0.310
Triglycerides	3	3	14.088	2	0.001	85.804	58	0.061
Bone mineral content	3	4	3.894	3	0.273	22.955	84	0.445
Bone mineral density	3	3	6.660	2	0.036	69.969	54	0.258
Bone speed of sound	3	10	73.374	9	<0.001	87.734	301	0.583
Anxiety	4	4	8.269	3	0.041	63.718	46	0.312
Depression	4	4	1.762	3	0.623	0.000	0	0.655
Physical self-concept	4	4	8.147	3	0.043	63.175	130	0.281

Notes: n number of studies, *k* number of effect sizes, *QB* Heterogeneity analysis statistics.

### 3.5 Publication bias analysis results

Table 1 also presents the publication bias analysis results. The Classic Fail-safe N tests demonstrated that the fail-safe numbers for the associations of speed performance with DBP, SBP, and depression all fell below their respective critical thresholds, indicating significant publication bias. Egger's test indicated that significant publication bias was present only in the association between speed performance and FFM (*p* < 0.05). Classic Fail-safe N and Egger's tests found no evidence of publication bias in other groups of associations. For the four groups of associations exhibiting publication bias, the Trim-and-Fill method was applied to adjust their pooled effect sizes.

### 3.6 Main effect analysis results

Table 2 presents the results of the main effect analysis.

**Table 2:** Results of the main effect analysis on the association between Speed Performance and health parameters.

Outcome Variable	n	*k*	Model	Effect size			Sensitivity analysis
				*r*	95%CI	*p*	
Body mass index	30	59	Random	0.179	0.154, 0.203	<0.001	0.172, 0.186
Fat-free mass	5	6	Random	−0.318	−0.504, −0.103	0.004	−0.353, −0.241
Fat mass	6	7	Random	0.247	0.151, 0.338	<0.001	0.212, 0.279
Percentage of body fat	11	24	Random	0.352	0.310, 0.393	<0.001	0.343, 0.369
Sum of skinfolds	5	20	Random	0.271	0.231, 0.310	<0.001	0.264, 0.282
Waist circumference	9	12	Random	0.211	0.146, 0.274	<0.001	0.192, 0.227
Diastolic blood pressure	3	3	Random	0.059	−0.024, 0.141	0.164	0.040, 0.106
Systolic blood pressure	3	3	Random	−0.019	−0.096, 0.058	0.630	−0.060, 0.024
Triglycerides	3	3	Random	0.191	0.082, 0.296	0.001	0.145, 0.239
Bone mineral content	3	4	Random	−0.382	−0.463, −0.294	<0.001	−0.425, −0.361
Bone mineral density	3	3	Random	−0.398	−0.539, −0.236	<0.001	−0.466, −0.332
Bone speed of sound	3	10	Random	−0.256	−0.374, −0.129	<0.001	−0.284, −0.215
Anxiety	4	4	Random	0.258	0.136, 0.373	<0.001	0.219, 0.306
Depression	4	4	Random	0.060	0.000, 0.119	0.049	0.038, 0.082
Physical self-concept	4	4	Random	−0.436	−0.542, −0.316	<0.001	−0.465, −0.375

*Notes:* n number of studies, *k* number of effect sizes.

As speed performance was assessed by time, with shorter duration indicating faster performance, a negative correlation signifies that faster performance is associated with higher outcome values, whereas a positive correlation reflects an association with lower outcome values.

Meta-analysis results indicate significant (*p* < 0.05) low-to-moderate correlations between speed performance and BMI (*r* = 0.179, 95%CI = 0.154 to 0.203), FFM (*r* = −0.318, 95%CI = −0.504 to −0.103), FM (*r* = 0.247, 95%CI = 0.151 to 0.338), BF% (*r* = 0.352, 95%CI = 0.310 to 0.393), SSF (*r* = 0.271, 95%CI = 0.231 to 0.310), WC (*r* = 0.211, 95%CI = 0.146 to 0.274), triglycerides (*r* = 0.191, 95%CI = 0.082 to 0.296), BMC (*r* = −0.382, 95%CI = −0.463 to −0.294), BMD (*r* = −0.398, 95%CI = −0.539 to −0.236), SoS (*r* = −0.256, 95%CI = −0.374 to −0.129), anxiety (*r* = 0.258, 95%CI = 0.136 to 0.373), and physical self-concept (*r* = −0.436, 95%CI = −0.542 to −0.316).

Among four groups of associations requiring Trim-and-Fill adjustment, the meta-analysis findings for the associations between speed performance with FFM (post-correction: *r* = −0.508, 95%CI = −0.647 to −0.337), DBP (post-correction: *r* = 0.059, 95%CI = −0.024 to 0.141), and SBP (post-correction: *r* = −0.071, 95%CI = −0.145 to 0.004) remained unchanged, indicating that publication bias was minimally affected. Conversely, meta-analysis findings for the association between speed performance and depression were unstable and liable to reversal (post-correction: *r* = 0.038, 95% CI = −0.012 to 0.088), indicating a need for further supporting studies.

### 3.7 Sensitivity analysis results

Table 2 also presents the sensitivity analysis results. Sensitivity analysis revealed that eliminating individual studies significantly altered results for DBP and SBP. This suggests unstable meta-analysis outcomes for both associations due to extreme effect sizes. Consequently, quantitative analysis (including moderator analysis) was abandoned, and a systematic review approach was only adopted for these two groups of associations.

Substantial heterogeneity was observed for FFM (*p* < 0.1, *I*^2^ = 98.172). After excluding the study by Liu XH et al.,^68^ heterogeneity significantly decreased (*p* = 0.094, *I*^2^ = 49.574), indicating this study as the primary heterogeneity source. Given that moderate heterogeneity persisted among the remaining studies, a random-effects model was retained. The analysis demonstrated stable results (*r* = −0.241, 95% CI = −0.317 to −0.163, *p* < 0.05), suggesting that the association between speed performance and FFM remained robust after exclusion of the identified study.

Moderate initial heterogeneity was observed for anxiety (*p* < 0.1, *I*^2^ = 63.718) and physical self-concept (*p* < 0.1, *I*^2^ = 63.175). Following the removal of the studies by García FG et al.^57^ for anxiety and Reigal-Garrido RE et al.^84^ for physical self-concept, heterogeneity was eliminated in both analyses (*p* > 0.1, *I*^2^ = 0.000), identifying these studies as primary heterogeneity sources. Potential reasons include differences in psychological questionnaires and types of speed tests. Given the elimination of statistical heterogeneity and the increased methodological homogeneity among the remaining studies, fixed-effect meta-analyses were conducted as a sensitivity analysis. The pooled effects for anxiety (*r* = 0.306, 95% CI = 0.234 to 0.375, *p* < 0.05) and physical self-concept (*r* = −0.375, 95% CI = −0.464 to −0.279, *p* < 0.05) remained statistically significant, supporting the robustness of the main findings.

For the other groups of associations examined, both the main effect analysis results and heterogeneity levels showed no substantial changes.

### 3.8 Moderator analysis results

Table 3, Table 4 present the moderator analysis results. All groups of associations that exhibit the high heterogeneity should undergo moderator analysis. However, insufficient effect sizes prevented moderator analyses for triglycerides, BMD, and physical self-concept.

**Table 3:** Results of the meta-regression analysis of moderator effects.

Outcome Variable	Moderator variable	β	SE	95%CI	z	*p*	*R*^2^
Body mass index	Average age	−0.0141	0.0039	−0.0217, −0.0066	−3.65	0.0003	0.46
	Publish year	−0.0071	0.0028	−0.0126, −0.0015	−2.49	0.0128	0.24
	Sample size	0.0000	0.0000	0.0000, 0.0000	0.16	0.8735	0.01
Percentage of body fat	Average age	0.0427	0.0079	0.0272, 0.0583	5.40	0.0000	0.70
	Publish year	0.0073	0.0067	−0.0058, 0.0203	1.09	0.2754	0.08
	Sample size	0.0001	0.0000	0.0000, 0.0002	2.67	0.0077	0.36
Sum of skinfolds	Average age	0.0167	0.0060	0.0049, 0.0286	2.78	0.0055	0.39
	Publish year	0.0028	0.0025	−0.0021, 0.0079	1.15	0.2518	0.00
	Sample size	−0.0000	0.0001	−0.0002, 0.0002	−0.00	0.9994	0.00

*Notes: R*^*2*^ proportion of explained variance.

**Table 4:** Results of the subgroup analysis of moderator effects.

Outcome Variable	Moderator variable	Heterogeneity			Subgroups	n	*k*	Effect size		
		*QB*	*df*	*p*				*r*	95%CI	*p*
Body mass index	Region	8.627	4	0.071	AFRO	1	8	0.430	0.210, 0.608	<0.001
					AMRO	3	3	0.207	0.096, 0.314	<0.001
					EMRO	1	2	0.138	−0.051, 0.317	0.152
					EURO	17	32	0.184	0.150, 0.219	<0.001
					WPRO	8	14	0.146	0.113, 0.178	<0.001
	Sex	6.503	1	0.011	Female	13	17	0.141	0.086, 0.195	<0.001
					Male	14	25	0.231	0.187, 0.274	<0.001
	Type of speed test	4.762	1	0.029	Sprint	17	43	0.165	0.135, 0.195	<0.001
					Speed shuttle run	13	16	0.209	0.184, 0.235	<0.001
Fat-free mass	Region	2.438	1	0.118	EURO	2	2	−0.208	−0.348, −0.059	0.006
					WPRO	2	3	−0.418	−0.603, −0.191	0.001
	Type of speed test	1.313	1	0.252	Sprint	3	4	−0.369	−0.571, −0.126	0.004
					Speed shuttle run	2	2	−0.208	−0.348, −0.059	0.006
Fat mass	Age group	0.093	1	0.760	6 < age ≤12	2	2	0.235	−0.085, −0.511	0.149
					12 < age ≤18	2	3	0.286	0.157, 0.406	<0.001
	Type of speed test	6.733	1	0.009	Sprint	3	3	0.147	0.067, 0.225	<0.001
					Speed shuttle run	3	4	0.317	0.215, 0.412	<0.001
Percentage of body fat	Region	5.141	3	0.162	AFRO	1	8	0.332	0.218, 0.436	<0.001
					EMRO	2	3	0.299	−0.022, 0.565	0.068
					EURO	6	10	0.392	0.355, 0.428	<0.001
					WPRO	1	2	0.244	0.095, 0.382	0.001
	Sex	25.849	1	<0.001	Female	2	2	0.102	−0.021, 0.221	0.103
					Male	5	16	0.407	0.378, 0.435	<0.001
	Type of speed test	0.019	1	0.890	Sprint	7	20	0.355	0.309, 0.400	<0.001
					Speed shuttle run	4	4	0.347	0.228, 0.456	<0.001
Sum of skinfolds	Region	3.824	1	0.051	AMRO	2	3	0.163	0.036, 0.285	0.012
					EURO	3	17	0.291	0.252, 0.328	<0.001
	Sex	0.177	1	0.674	Female	4	17	0.270	0.225, 0.314	<0.001
					Male	2	2	0.318	0.088, 0.516	0.007
	Type of speed test	3.225	1	0.073	Sprint	1	2	0.119	0.036, 0.200	0.005
					Speed shuttle run	4	18	0.286	0.250, 0.321	<0.001
	Type of outcome measure	3.450	2	0.178	Sum of 2 skinfolds	1	2	0.119	0.036, 0.200	0.005
					Sum of 5 skinfolds	2	15	0.287	0.248, 0.325	<0.001
					Sum of 6 skinfolds	1	2	0.329	0.108, 0.518	0.004
Waist circumference	Age group	0.923	2	0.630	3 ≤ age ≤6	2	3	0.151	0.010, 0.286	0.036
					6 < age ≤12	4	5	0.203	0.095, 0.307	<0.001
					12 < age ≤19	1	2	0.265	0.067, 0.442	0.009
	Region	2.222	2	0.329	EMRO	1	2	0.094	−0.099, 0.281	0.339
					EURO	6	7	0.245	0.140, 0.344	<0.001
					WPRO	1	2	0.175	0.106, 0.243	<0.001
	Sex	3.963	1	0.047	Female	3	3	0.104	0.006, 0.200	0.037
					Male	3	3	0.238	0.148, 0.323	<0.001
	Type of speed test	2.428	1	0.119	Sprint	4	6	0.169	0.068, 0.267	0.001
					Speed shuttle run	5	6	0.254	0.212, 0.295	<0.001
Bone speed of sound	Age group	10.741	1	0.001	3 ≤ age ≤6	1	2	−0.019	−0.133, 0.096	0.745
					6 < age ≤19	2	8	−0.311	−0.431, −0.180	<0.001
	Sex	4.189	1	0.041	Female	3	5	−0.141	−0.231, −0.048	0.003
					Male	3	5	−0.374	−0.547, −0.170	<0.001
Anxiety	Type of speed test	1.994	1	0.158	Sprint	2	2	0.203	0.098, 0.303	<0.001
					Speed shuttle run	2	2	0.338	0.245, 0.425	<0.001

*Notes: AFRO* the African Region*, AMRO* the Region of the Americas*, EURO* the European Region*, EMRO* the Eastern Mediterranean Region*, WPRO* the Western Pacific Region*,* n number of studies*, k* number of effect sizes, *QB* Heterogeneity analysis statistics, Subgroup analysis by age was performed only when meta-regression was not feasible, excluding studies that spanned multiple age ranges.

BMI: Average age, publication year, sex, and type of speed test all showed significant moderator effects (*p* < 0.05), whereas region did not (*p* > 0.05).

FM: Type of speed test exhibited a significant moderator effect (*p* < 0.05), whereas age group did not (*p* > 0.05).

BF%: Average age, sample size, and sex were identified as significant moderators (*p* < 0.05), whereas region, type of speed test, and publication year were not (*p* > 0.05).

SSF: Average age exhibited a significant positive moderator effect (*p* < 0.05), whereas type of outcome measure, publication year, region, sample size, sex, and type of speed test were not significant moderators (*p* > 0.05).

WC: Sex was a significant moderator (*p* < 0.05), whereas age group, region, and type of speed test were not (*p* > 0.05).

SoS: Age group and sex exhibited significant moderator effects (*p* < 0.05).

FFM: No potential moderator showed significant effects (*p* > 0.05).

Anxiety: No potential moderator showed significant effects (*p* > 0.05).

### 3.9 Certainty of evidence

The results demonstrate that the certainty of evidence for BMI, FM, BF%, WC, and BMC was evaluated as ‘low’, whereas the certainty of evidence for FFM, SSF, DBP, SBP, triglycerides, BMD, SoS, anxiety, depression, and physical self-concept was evaluated as ‘very low’ (Supplementary material, Table S4).

## 4 Discussion

### 4.1 Speed performance and health benefits

This meta-analysis demonstrates that speed performance is significantly associated with multiple dimensions of health in children and adolescents. Moderator analyses indicated that the strength of these associations varies according to demographic and methodological characteristics. Importantly, across these diverse health domains, speed performance should not be interpreted as entirely independent of other fitness components. Rather, it likely shares underlying neuromuscular and metabolic determinants with capacities such as muscular strength and cardiorespiratory fitness. Consequently, the observed associations may partially reflect these shared physiological mechanisms rather than effects unique to speed performance alone.

#### 4.1.1 Anthropometric and adiposity parameters

Our findings show that significant correlations exist between speed performance and anthropometric and adiposity parameters. From a biomechanical perspective, increased body mass necessitates higher propulsive forces to achieve the same acceleration. Overweight/obese children and adolescents require greater force to overcome gravity and propel their bodies during speed tests compared to their normal-weight counterparts. A previous meta-analysis revealed a negative association between muscular fitness and adiposity, implying that increased body fat may not improve the ability to generate force.^13^ Underweight children and adolescents have been observed to have a lower proportion of type IIb (fast-twitch) muscle fibers, which rely predominantly on the anaerobic energy system and are crucial for speed performance.^95^ This physiological characteristic limits their capacity to generate the explosive force required for optimal results. Collectively, this suggests that abnormal body composition (whether above or below the normal range) may impair speed performance.

Better speed performance may be indicative of more favorable anthropometric and adiposity profiles, possibly through increased energy expenditure. It is closely related to the skeletal muscle's capacity to rapidly generate force, and thus superior performance may reflect positive adaptations in the underlying metabolic, structural, and functional characteristics of muscle. Given that skeletal muscle is a metabolically active tissue contributing significantly to basal metabolic rate,^96^ these adaptations may increase total daily energy expenditure.

Meta-regression analysis demonstrated that with increasing average age, the association between speed performance and BMI gradually weakened, while the association with both BF% and SSF progressively strengthened. This divergence highlights the limitations of BMI as a measure of body composition, as it cannot differentiate between fat mass and fat-free mass. During child and adolescent development, the normative age-related increase in BMI primarily reflects the accumulation of fat-free mass, which may benefit speed performance.^97^ In contrast, BF% and SSF more accurately indicate overweight or obese status, and thus better capture the negative effects of additional body fat on speed performance.^98^ Additionally, the correlation between speed performance and BMI weakened over more recent publication years. This pattern may reflect secular trends in children and adolescents, where declining speed performance^99^, ^100^, ^101^ is accompanied by increasing BMI.^102^ The reduction in variability of both variables may have led to clustering of measurements at the extremes, which diminished the strength of their association. Furthermore, sample size significantly moderated the association between speed performance and BF%, as larger sample sizes better capture population heterogeneity, enabling more accurate assessment of correlations between variables.

Subgroup analysis revealed that sex significantly moderated the associations of speed performance with BMI, BF%, and WC, with consistently stronger correlations observed in males. These differences likely stem from sex-based differences in factors such as physical activity intensity and essential fat, among others.^103^ Speed shuttle runs demonstrated stronger associations with BMI and FM than sprints, likely due to their unique change-of-direction component, which requires rapid deceleration followed by immediate re-acceleration. Consequently, the detrimental effects of additional body fat appear more pronounced in males than in females, and in speed shuttle runs than in sprints.

#### 4.1.2 Cardiometabolic parameters

Our meta-analysis revealed an inconclusive association between speed performance and blood pressure, whereas a significant association was observed for triglycerides.

Large-scale cross-sectional data from Chinese children and adolescents indicate significant associations between speed performance and blood pressure,^104^ with additional evidence suggesting that it predicts cardiometabolic risk and partially mediates the correlation between obesity and cardiometabolic health.^105^

Taken together, although evidence for specific cardiometabolic outcomes remains inconsistent, prior research, alongside the associations observed in the present meta-analysis between speed performance and anthropometric and adiposity parameters, which are important cardiometabolic risk factors, provides indirect support for a potential correlation between speed performance and cardiometabolic health. This relationship may be explained by several plausible pathways. Speed performance has been shown to correlate with both objectively measured and self-reported moderate-to-vigorous physical activity,^106^ which is well established to confer cardiometabolic benefits.^107^ In addition, speed performance is closely related to muscular fitness,^108^ suggesting that its association with cardiometabolic health may be mediated by biological mechanisms similar to those underlying muscular fitness,^11^ such as reduced adipose tissue^77^ and improved insulin sensitivity.^109^

#### 4.1.3 Bone parameters

Our meta-analysis identified a significant negative correlation between speed performance and bone parameters. These findings are supported by several studies. Gracia-Marco et al. reported that adolescents with better speed performance had higher BMC than their slower counterparts.^110^ Additionally, three longitudinal studies with follow-up periods of 15 years,^111^ 20 years,^112^ and 27 years^113^ all revealed that better speed performance during childhood and adolescence was associated with higher levels of BMC and BMD later in life. High-intensity running is commonly incorporated in speed tests. Previous network meta-analysis suggested that exercises based on high-intensity running are effective in enhancing speed performance.^114^ Children and adolescents who regularly engage in such speed-oriented training demonstrate superior performance in various speed assessments.^115^

According to Newton's Third Law of Motion, the force and reaction between two interacting bodies are always equal in magnitude and opposite in direction, acting in the same straight line. This implies that maximal running speed requires optimal ground reaction force. While running at a high intensity, children and adolescents exert a force on the ground ranging from two to three times their body weight.^116^ A larger ground reaction force during physical activity may lead to a more powerful osteogenic stimulus, which is more beneficial for bone health^.^^117^ Three review studies provide supporting evidence, finding that high-impact vigorous physical activities relying on the anaerobic energy system can significantly improve bone health.^118^, ^119^, ^120^ This may help to explain why speed performance was associated with bone parameters.

Subgroup analyses demonstrated that age group and sex significantly moderated the association between speed performance and SoS, with stronger correlations observed in school-aged children than in preschool children, and in males than in females. These patterns may stem from age- and sex-based differences in growth hormone, sex hormone, and physical activity, all of which contribute to bone development.^121^

#### 4.1.4 Psychological parameters

Our meta-analysis identified a significant correlation between speed performance and both physical self-concept and anxiety. These findings are supported by several studies. An intervention study showed that improved speed performance was associated with decreased anxiety and increased self-confidence.^122^ Furthermore, conclusions from cross-sectional studies support that speed performance correlates with self-confidence^123^ and self-esteem.^124^

Electroencephalogram (EEG) patterns reflect neuronal activity in the cerebral cortex, which can indicate the functional state of the central nervous system (CNS). A study identified a significant association between speed performance and specific EEG parameters among children.^125^ Within the CNS, the hippocampus, amygdala, and prefrontal cortex function as integral parts of the limbic system and play crucial roles in regulating the hypothalamic-pituitary-adrenal axis. As the body's primary stress hormone system, this axis profoundly impacts psychological health.

Furthermore, the correlation between speed performance and psychological health may also be explained by psychosocial mechanisms. A recent meta-analysis revealed that children and adolescents with better psychological health generally exhibit higher levels of physical activity.^126^ This is partly attributed to their increased motivation for physical activity, which further enhances their physical performance. However, more research is needed to investigate the mechanisms underlying the association between speed performance and psychological health.

### 4.2 Practical implications

Our findings suggest important practical implications for fitness assessment and health promotion in children and adolescents. Speed performance assessment is already integral to several widely implemented fitness test batteries and national surveillance systems, such as ALPHA,^6^ PREFIT,^7^ the German Motor Test^127^ and the Chinese National Student Physical Fitness Standard. However, the recently proposed Youth Fitness International Test (YFIT) battery does not include speed performance among its core tests.^128^ This exclusion likely reflects a prioritization of fitness components with the strongest evidence regarding current health status and predictive value for future health outcomes, such as cardiorespiratory fitness and muscular fitness. Within this context, the present meta-analysis provides complementary quantitative evidence indicating that speed performance is meaningfully associated with multiple health-related parameters. These findings suggest that speed performance may have additional value as a component of fitness assessment in children and adolescents, particularly in applied or educational settings.

From a feasibility perspective, speed tests are time-efficient, low-cost, and easy to administer in school environments. Moreover, tasks assessing speed performance align closely with the spontaneous, intermittent, and high-intensity movement patterns typical of children's and adolescents' daily activity.^5^ Therefore, speed may serve as a useful adjunct to established HRF batteries, helping to contextualize standardized testing within the real-world movement behaviors of this population. However, given the predominantly observational nature of the included evidence, speed performance should be interpreted primarily as a marker of health and neuromuscular function rather than a direct intervention target.

### 4.3 Strengths and limitations

To the best of our knowledge, this is the first systematic review and meta-analysis to provide a comprehensive quantitative synthesis of the associations between speed performance and multiple health-related parameters in children and adolescents. This work is strengthened by a large aggregated sample size across outcomes and the application of moderator analyses, which enabled the exploration of potential sources of heterogeneity and provided a more nuanced interpretation of the findings across study characteristics.

Several limitations warrant cautious interpretation of these findings. First, the available evidence was predominantly cross-sectional, precluding causal inference and limiting conclusions regarding directionality. Second, substantial heterogeneity was observed in several pooled analyses, likely reflecting variability in participant characteristics, speed assessment methods, and health outcome measurements. Third, the RoB varied across studies, with the certainty of some pooled estimates downgraded due to imprecision. Fourth, studies reporting only regression-based estimates without accessible correlation coefficients were excluded, which may have resulted in the loss of eligible evidence and introduced potential selection bias. Fifth, none of the included studies reported analyses that adjusted for other HRF fitness components, such as cardiorespiratory fitness or muscular fitness. Consequently, the findings reflect overall associations, and the independent contribution of speed performance to health outcomes remains to be fully elucidated. Additionally, the exclusion of special populations (e.g., athletes or people with chronic or acute illnesses or injuries), while intended to ensure sample homogeneity, may preclude the examination of potential differential associations across diverse population types. Finally, although comprehensive searches were conducted, publication and selection bias cannot be entirely ruled out.

## 5 Conclusion

Our findings indicate that better speed performance is associated with favorable anthropometric and adiposity profiles (i.e., lower BMI, BF%, FM, SSF, and WC; higher FFM), better bone health (i.e., higher BMC, BMD, and SoS), and positive psychological outcomes (i.e., lower anxiety and higher physical self-concept). These associations were moderated by relevant factors (e.g., age, publication year, region, sample size, sex, and speed assessment methods), with moderating patterns differing across specific health outcomes. In contrast, evidence regarding associations with blood pressure and depression was inconclusive. Overall, the low-to-moderate associations observed across a wide range of health outcomes suggest that speed performance may represent a meaningful marker of health in children and adolescents. Given its simplicity, feasibility, and low cost, speed performance assessment may serve as a practical tool for large-scale health monitoring and screening in pediatric populations. Future longitudinal and intervention studies are warranted to provide further insights into the impact of speed performance on health in children and adolescents.

## Confirmation of ethical compliance

Not applicable.

## Funding information

This work is funded by the National Social Science Fund of China (22BTY098).

## Competing interest

Author Jane Jie Yu is a member of the Editorial Board of *Journal of Exercise Science and Fitness*. Jane Jie Yu was not involved in the journal's peer review process of, or decisions related to, this manuscript.

## Data Availability

All data generated or analyzed during this study are included in this published article and its supplementary information files.
