# Supplementary material for: The associations between speed performance and health outcomes in children and adolescents: A systematic review and meta-analysis
Source: J Exerc Sci Fit. 2026 Feb 26;24(2):200461. doi: 10.1016/j.jesf.2026.200461 (PMC12989721; doi:10.1016/j.jesf.2026.200461)
Supplement: Multimedia component 1 [file mmc1.docx]

**Supplementary Material**

**The catalogue of supplementary material**

[Table S1: PRISMA item checklist for systematic reviews 2](#_Toc585330530)

[Table S2: Summary of characteristics of all the included studies 6](#_Toc27630803)

[Table S3: The risk of bias assessment of the included studies 13](#_Toc534438269)

[Table S4: GRADE summary of findings 16](#_Toc1527375329)

[Figure S1: Forest plot evaluating the association between speed performance and body mass index 17](#_Toc1725121912)

[Figure S2: Forest plot evaluating the association between speed performance and fat-free mass 18](#_Toc947256837)

[Figure S3: Forest plot evaluating the association between speed performance and fat mass 18](#_Toc1249384248)

[Figure S4: Forest plot evaluating the association between speed performance and percentage of body fat 19](#_Toc305955770)

[Figure S5: Forest plot evaluating the association between speed performance and sum of skinfolds 20](#_Toc1122775472)

[Figure S6: Forest plot evaluating the association between speed performance and waist circumference 21](#_Toc548551715)

[Figure S7: Forest plot evaluating the association between speed performance and diastolic blood pressure 21](#_Toc361377434)

[Figure S8: Forest plot evaluating the association between speed performance and systolic blood pressure 22](#_Toc586779522)

[Figure S9: Forest plot evaluating the association between speed performance and triglycerides 22](#_Toc758519230)

[Figure S10: Forest plot evaluating the association between speed performance and bone mineral content 23](#_Toc969770018)

[Figure S11: Forest plot evaluating the association between speed performance and bone mineral density 23](#_Toc1671295443)

[Figure S12: Forest plot evaluating the association between speed performance and bone speed of sound​ 24](#_Toc376407741)

[Figure S13: Forest plot evaluating the association between speed performance and anxiety 24](#_Toc1945562572)

[Figure S14: Forest plot evaluating the association between speed performance and depression 25](#_Toc1484138382)

[Figure S15: Forest plot evaluating the association between speed performance and physical self-concept 25](#_Toc891226369)

[Search strategy 26](#_Toc143145958)

# Table S1: PRISMA item checklist for systematic reviews

| **Section and Topic** | **Item** | **Checklist item** | **Location where item is reported** |
| --- | --- | --- | --- |
| **TITLE** | | |  |
| Title | 1 | Identify the report as a systematic review. | Title page |
| **ABSTRACT** | | |  |
| Abstract | 2 | See the PRISMA 2020 for Abstracts checklist. | Abstract |
| **INTRODUCTION** | | |  |
| Rationale | 3 | Describe the rationale for the review in the context of existing knowledge. | Introduction |
| Objectives | 4 | Provide an explicit statement of the objective(s) or question(s) the review addresses. | Introduction |
| **METHODS** | | |  |
| Eligibility criteria | 5 | Specify the inclusion and exclusion criteria for the review and how studies were grouped for the syntheses. | Methods: Eligibility criteria |
| Information sources | 6 | Specify all databases, registers, websites, organisations, reference lists and other sources searched or consulted to identify studies. Specify the date when each source was last searched or consulted. | Methods:  Data Sources and Search Strategy |
| Search strategy | 7 | Present the full search strategies for all databases, registers and websites, including any filters and limits used. | Supplementary Material |
| Selection process | 8 | Specify the methods used to decide whether a study met the inclusion criteria of the review, including how many reviewers screened each record and each report retrieved, whether they worked independently, and if applicable, details of automation tools used in the process. | Methods: Study selection and data extraction |
| Data collection process | 9 | Specify the methods used to collect data from reports, including how many reviewers collected data from each report, whether they worked independently, any processes for obtaining or confirming data from study investigators, and if applicable, details of automation tools used in the process. | Methods: Study selection and data extraction |
| Data items | 10a | List and define all outcomes for which data were sought. Specify whether all results that were compatible with each outcome domain in each study were sought (e.g. for all measures, time points, analyses), and if not, the methods used to decide which results to collect. | Methods: Study selection and data extraction |
|  | 10b | List and define all other variables for which data were sought (e.g. participant and intervention characteristics, funding sources). Describe any assumptions made about any missing or unclear information. | Methods: Study selection and data extraction |
| Study risk of bias assessment | 11 | Specify the methods used to assess risk of bias in the included studies, including details of the tool(s) used, how many reviewers assessed each study and whether they worked independently, and if applicable, details of automation tools used in the process. | Methods: Study selection and data extraction |
| Effect measures | 12 | Specify for each outcome the effect measure(s) (e.g. risk ratio, mean difference) used in the synthesis or presentation of results. | Methods: Study selection and data extraction |
| Synthesis methods | 13a | Describe the processes used to decide which studies were eligible for each synthesis (e.g. tabulating the study intervention characteristics and comparing against the planned groups for each synthesis (item #5)). | Methods: Data synthesis and meta-analysis |
|  | 13b | Describe any methods required to prepare the data for presentation or synthesis, such as handling of missing summary statistics, or data conversions. | Methods: Data synthesis and meta-analysis |
|  | 13c | Describe any methods used to tabulate or visually display results of individual studies and syntheses. | Methods: Data synthesis and meta-analysis |
|  | 13d | Describe any methods used to synthesize results and provide a rationale for the choice(s). If meta-analysis was performed, describe the model(s), method(s) to identify the presence and extent of statistical heterogeneity, and software package(s) used. | Methods: Data synthesis and meta-analysis |
|  | 13e | Describe any methods used to explore possible causes of heterogeneity among study results (e.g. subgroup analysis, meta-regression). | Methods: Data synthesis and meta-analysis |
|  | 13f | Describe any sensitivity analyses conducted to assess robustness of the synthesized results. | Methods: Data synthesis and meta-analysis |
| Reporting bias assessment | 14 | Describe any methods used to assess risk of bias due to missing results in a synthesis (arising from reporting biases). | Methods: Risk of bias assessment |
| Certainty assessment | 15 | Describe any methods used to assess certainty (or confidence) in the body of evidence for an outcome. | Methods: Certainty of evidence assessment |
| **RESULTS** | | |  |
| Study selection | 16a | Describe the results of the search and selection process, from the number of records identified in the search to the number of studies included in the review, ideally using a flow diagram. | Results |
|  | 16b | Cite studies that might appear to meet the inclusion criteria, but which were excluded, and explain why they were excluded. | Results |
| Study characteristics | 17 | Cite each included study and present its characteristics. | Results |
| Risk of bias in studies | 18 | Present assessments of risk of bias for each included study. | Supplementary Material |
| Results of individual studies | 19 | For all outcomes, present, for each study: (a) summary statistics for each group (where appropriate) and (b) an effect estimate and its precision (e.g. confidence/credible interval), ideally using structured tables or plots. | Results |
| Results of syntheses | 20a | For each synthesis, briefly summarise the characteristics and risk of bias among contributing studies. | Results |
|  | 20b | Present results of all statistical syntheses conducted. If meta-analysis was done, present for each the summary estimate and its precision (e.g. confidence/credible interval) and measures of statistical heterogeneity. If comparing groups, describe the direction of the effect. | Results |
|  | 20c | Present results of all investigations of possible causes of heterogeneity among study results. | Results |
|  | 20d | Present results of all sensitivity analyses conducted to assess the robustness of the synthesized results. | Supplementary Material |
| Reporting biases | 21 | Present assessments of risk of bias due to missing results (arising from reporting biases) for each synthesis assessed. | Results, |
| Certainty of evidence | 22 | Present assessments of certainty (or confidence) in the body of evidence for each outcome assessed. | Results, Supplementary Material |
| **DISCUSSION** | | |  |
| Discussion | 23a | Provide a general interpretation of the results in the context of other evidence. | Discussion |
|  | 23b | Discuss any limitations of the evidence included in the review. | Strength and limitations |
|  | 23c | Discuss any limitations of the review processes used. | Strength and limitations |
|  | 23d | Discuss implications of the results for practice, policy, and future research. | Strength and limitations |
| **OTHER INFORMATION** | | |  |
| Registration and protocol | 24a | Provide registration information for the review, including register name and registration number, or state that the review was not registered. | Abstract, Methods |
|  | 24b | Indicate where the review protocol can be accessed, or state that a protocol was not prepared. | Methods |
|  | 24c | Describe and explain any amendments to information provided at registration or in the protocol. | Methods |
| Support | 25 | Describe sources of financial or non-financial support for the review, and the role of the funders or sponsors in the review. | Funding |
| Competing interests | 26 | Declare any competing interests of review authors. | Declaration of competing interest |
| Availability of data, code and other materials | 27 | Report which of the following are publicly available and where they can be found: template data collection forms; data extracted from included studies; data used for all analyses; analytic code; any other materials used in the review. | Supplementary information |

# Table S2: Summary of characteristics of all the included studies

| Author(s), date, country | Sample(M/F) | Age (year) | Speed Test | Outcomes of interest | Findings |
| --- | --- | --- | --- | --- | --- |
| Agha-Alinejad H et al., 2015, Iran | n=381(190/191) | 5-6 | 20 m sprint | BF%; BMI; WC | boy + BF%: *r* = 0.255***; girl + BF%: *r* = 0.045;  boy + BMI: *r* = 0.231***; girl + BMI: *r* = 0.042;  boy + WC: *r* = 0.187**; girl + WC: *r* = -0.009 |
| Andrade S et al., 2014, Ecuador | n=648(339/309) | 13.6±1.2 | 10×5 m SSR | TG | 10×5 m SSR + TG: *r* = 0.197 |
| Ara I et al., 2004, Spain | n=114(114/0) | 9.4±1.5 | 30 m sprint | BF%; FM | 30 m + BF%: *r* = 0.36*; 30 m + FM: *r* = 0.29* |
| Arriscado D et al., 2014, Spain | n=329(168/161) | 11.7±0.4 | 4×10 m SSR | BF%; BMI; WC;  DBP; SBP | 4×10 m SSR + BF%: *r* = 0.385**; 4×10 m SSR + BMI: *r* = 0.221**;  4×10 m SSR + WC: *r* = 0.273**; 4×10 m SSR + DBP: *r* = 0.105;  4×10 m SSR + SBP: *r* = -0.009 |
| Baj-Korpak J et al., 2022, Poland | n=703(266/437) | 12.21±1.11 | 3×10 m SSR | BMI | 3×10 m SSR + BMI: *r* = 0.120*** |
| Batez M et al., 2021, Serbia | n=130(51/79) | 8.60±0.61 | 10×5 m SSR | BMI | 10×5m SSR + BMI: r = 0.19* |
| Becerra et al., 2013, Spain | n=264(130/134) | 15.1±0.8 | 50 m sprint | Anxiety; Depression | 50 m + anxiety: *r* = 0.26***; 50 m + depression: *r* = -0.01 |
| Borrego-Balsalobre FJ et al., 2022, Spain | n=138(67/71) | 11.21±0.41 | 20 m sprint | BMI | 20 m + BMI: r = 0.367* |
| Carraro A et al., 2010, Italy | n=103(50/53) | 13.2 ± 0.7 | 10×5 m SSR | Physical self-concept | 10×5 m SSR + physical self-concept: *r* = −0.33** |
| Chen C et al., 1994, China | n=210(105/105) | 7-11 | 50 m sprint | FFM | boy + FFM: *r* = -0.240*; girl + FFM: *r* = -0.396** |
| Colella D et al., 2009, Italy | n=210(105/105) | 9.2 ± 1.1 | 20 m sprint | BMI | boy(8y) + BMI: *r* = 0.60**; girl(8y) + BMI: *r* = 0.46**;  boy(9y) + BMI: *r* = 0.32; girl(9y) + BMI: *r* = 0.14;  boy(10y) + BMI: *r* = 0.41*; girl(10y) + BMI: *r* = 0.22; |

Table S2 (continued)

| Author(s), date, country | Sample(M/F) | Age (year) | Speed Test | Outcomes of interest | Findings |
| --- | --- | --- | --- | --- | --- |
| Du J et al., 2011, China | n=1,100(550/550) | 7-18 | 50 m sprint | BMI | 50 m + BMI: *r* = 0.17** |
| Esmaeilzadeh S et al., 2015, Iran | n=456(456/0) | 9.3±1.2 | 30 m sprint | Depression | 30 m + depression: *r* = 0.09 |
| García FG, Gómez MR et al., 2011, Spain | n=78(36/42) | 11.4±0.7 | 50 m sprint | Anxiety | 4×10 m SSR + anxiety: *r* = -0.006 |
| García-Sánchez A et al., 2013, Spain | n=69(42/27) | 14.68 ± 1.36 | 4×10 m SSR | Physical self-concept | 4×10 m SSR + physical self-concept: *r* = −0.428** |
| Gonzalez-Suarez CB et al., 2013, Philippines | n=1,021(513/508) | 11.1±0.9 | 40 m sprint | BMI; WC | boy + BMI: *r* = 0.25***; girl + BMI: *r* = 0.14***;  boy + WC: *r* = 0.21***; girl + WC: *r* = 0.14*** |
| Gómez-Bruton A et al., 2020, Spain | n=92(50/42) | 4.81 ± 0.76 | 4×10 m SSR | BMC; BMD | 4×10 m SSR + BMC: *r* = -0.464**; 4×10 m SSR + BMD: *r* = -0.217** |
| Henriksson P et al., 2022, Sweden | n=411(211/200) | 9.5 ± 0.1 | 4×10 m SSR | BF%; BMI; FM;  FFM; DBP; SBP;  TG | 4×10 m SSR + BF%: *r* = 0.415**; 4×10 m SSR + BMI: *r* = 0.235**;  4×10 m SSR + FM: *r* = 0.384**; 4×10 m SSR + FFM: *r* = -0.139*;  4×10 m SSR + DBP: *r* = 0.106*; 4×10 m SSR + SBP: *r* = 0.051;  4×10 m SSR + TG: *r* = 0.289*** |
| Henriques-Neto D et al., 2020a, Portugal | n=366(129/237) | boy:14.09 ± 2.4  girl:15.10 ± 2.3 | 40 m sprint | T-SoS; R-SoS | boy + T-SOS: *r* = -0.588**; girl + T-SOS: *r* = -0.264**;  boy + R-SOS: *r* = -0.321**; girl + R-SOS: *r* = -0.091 |
| Henriques-Neto D et al., 2020b, Portugal | n=412(191/221) | 14.59 ± 2.36 | 20 m sprint | T-SoS; R-SoS | boy + T-SOS: *r* = -0.540**; girl + T-SOS: *r* = -0.201**;  boy + R-SOS: *r* = -0.293**; girl + R-SOS: *r* = -0.130 |
| Hensley LD et al., 1982, America | n=563(289/274) | boy:8.42 ± 1.12  girl:8.33 ± 1.03 | 40 yard sprint | SSF | boy + SSF: *r* = 0.21; girl + SSF: *r* = 0.02 |

Table S2 (continued)

| Author(s), date, country | Sample(M/F) | Age (year) | Speed Test | Outcomes of interest | Findings |
| --- | --- | --- | --- | --- | --- |
| Hernández-Jaña S et al., 2021, Chile | n=1,196(606/590) | 11.71 ± 1.06 | 4×10 m SSR | BMI; SSF | 4×10 m + BMI: *r* = 0.208*; 4×10 m + SSF: *r* = 0.236* |
| Klein M et al., 2013, German | n=839(437/402) | 6-18 | 20 m sprint | BMI | boy + BMI: *r* = 0.31***; girl + BMI: *r* = 0.07 |
| Kurtoğlu A et al., 2024, Turkey | n=282(130/152) | boy:8.26±1.84  girl:8.74 ± 1.83 | 20 m sprint | WC | 20m + WC: *r* = 0.40* |
| Kwieciński J et al., 2018, Poland | n_1_=507(275/232)  n_2_=767(440/327)  n_3_=868(524/344) | n_1_=13  n_2_=14  n_3_=15 | 50 m sprint | BMI | boy(13y) + BMI: *r* = 0.135*; girl(13y) + BMI: *r* = 0.008;  boy(14y) + BMI: *r* = 0.033; girl(14y) + BMI: *r* = 0.025;  boy(15y) + BMI: *r* = -0.075; girl(15y) + BMI: *r* = 0.198*** |
| Liu XH et al., 2018, China | n=4,917(2,486/2,431) | 7-18 | 50 m sprint | FM; FFM | 50 m + FM: *r* = 0.152**; 50 m + FFM: *r* = -0.560** |
| Llagjeviq-Govori A et al., 2025, North Macedonia | n=2,197(1,096/1,101) | 8.1±1.3 | 4×10 m SSR | BMI | boy + BMI: *r* = 0.256***; girl+ BMI: *r* = 0.192*** |
| López Alonzo SJ et al., 2022, Mexico | n=531(260/271) | 13.01 ± 0.9 | 4×10 m SSR | BMI; WC | 4×10 m SSR + BMI: *r* = 0.314**; 4×10 m SSR + WC: *r* = 0.270** |
| López-Gil JF et al., 2020, Spain | n=370(204/166) | 8.7±1.8 | 4×10 m SSR | BF%; BMI; WC | 4×10 m SSR + BF%: *r* = 0.401**; 4×10 m SSR + BMI: *r* = 0.263**;  4×10 m SSR + WC: *r* = 0.214** |
| Madi DM et al., 2018, Serbia | n=296(184/112) | boy:5.89 ± 1.02  girl:5.82 ± 1.04 | 20 m sprint | C-SoS | boy + C-SoS: *r* = -0.057; girl + C-SoS: *r* = 0.044 |

Table S2 (continued)

| Author(s), date, country | Sample(M/F) | Age (year) | Speed Test | Outcomes of interest | Findings |
| --- | --- | --- | --- | --- | --- |
| Malina RM et al., 2018, Belgium | n=6,455(0/6,455) | 7-17 | 10×5 m SSR | SSF | 7y + SSF: *r* = 0.14*; 8y + SSF: *r* = 0.30*; 9y + SSF: *r* = 0.16*;  10y + SSF: *r* = 0.23*; 11y + SSF: *r* = 0.34*; 12y + SSF: *r* = 0.35*;  13y + SSF: *r* = 0.29*; 14y + SSF: *r* = 0.27*; 15y + SSF: *r* = 0.35*;  16y + SSF: *r* = 0.30*; 17y + SSF: *r* = 0.34* |
| Martinez‐Tellez B et al., 2016, Spain | n=403(233/170） | 3-5 | 4×10 m SSR | BMI; WC | 4×10 m SSR + BMI: *r* = 0.168*; 4×10 m SSR + WC: *r* = 0.246*** |
| Mello JB et al., 2022, Brazil | n=160(85/75) | 8.90 ± 1.56 | 20 m sprint | BMC; BMD | 20 m + BMC: *r* = -0.434**; 20 m + BMD: *r* = -0.420** |
| Mello JB et al., 2023, Brazil | n=160(85/75) | 8.90 ± 1.50 | 20 m sprint | BMD | 20 m + BMD: *r* = −0.510*** |
| Mendoza-Muñoz M et al., 2020, Spain | n=225(108/117) | 13.00±1.0 | 4×10 m SSR | BF%; BMI; FM;  FFM | 4×10 m SSR + BF%: *r* = 0.406**; 4×10 m SSR + BMI: *r* = 0.093;  4×10 m SSR + FM: *r* = 0.232**; 4×10 m SSR + FFM: *r* = -0.287** |
| Moliner-Urdiales D et al., 2011, Spain | n=310(153/157) | boy:14.8 ± 1.3  girl:14.8 ± 1.1 | 4×10 m SSR | FM; SSF; WC | boy + FM: *r* = 0.415***; girl + FM: *r* = 0.212**;  boy + SSF: *r* = 0.430***; girl + SSF: *r* = 0.219**;  boy +WC: *r* = 0.359***; girl + WC: *r* = 0.166* |
| Moradi A et al., 2019, Iran | n=206(206/0) | 11.0±0.8 | 30 m sprint | BF% | 30 m + BF%: *r* = 0.55** |
| Morano M et al., 2011, Italy | n=260(140/120) | 12.2±0.9 | 30 m sprint | BMI | boy + BMI: *r* = 0.48***; girl + BMI: *r* = 0.50*** |
| Moura‐Dos‐Santos MA et al., 2015, Brazil | n=483(251/232) | 8.78 ± 1.0 | 20 m sprint | BF%; BMI; FFM | 20 m + BF%: *r* = 0.2787***; 20 m + BMI: *r* = 0.0932;  20 m + FFM: *r* = -0.2309*** |

Table S2 (continued)

| Author(s), date, country | Sample(M/F) | Age (year) | Speed Test | Outcomes of interest | Findings |
| --- | --- | --- | --- | --- | --- |
| Muhumbe E et al., 2014, South African | n=325(325/0) | 7-10 | 35 m sprint | BF%; BMI | 7y + rural + BF%: *r* = 0.3025***; 7y + urban + BF%: *r* = 0.2507***;  7y + rural + BMI: *r* = 0.2714**; 7y + urban + BMI: *r* = 0.0419;  8y + rural + BF%: *r* = 0.2507*; 8y + urban + BF%: *r* = 0.4056**;  8y + rural + BMI: *r* = 0.3025**; 8y + urban + BMI: *r* = 0.1674;  9y + rural + BF%: *r* = 0.3748***; 9y + urban + BF%: *r* = 0.3748***;  9y + rural + BMI: *r* = 0.2818***; 9y + urban + BMI: *r* = 0.6081***;  10y + rural + BF%: *r* = 0.3645***; 10y + urban + BF%: *r* = 0.3953***;  10y + rural + BMI: *r* = 0.7847***; 10y + urban + BMI: *r* = 0.7943*** |
| Qiu S et al., 2024, China | n=23,191(11,559/11,632) | 7-18 | 50 m sprint | BMI | boy + BMI: *r* =0.0701**; girl + BMI: *r* = 0.0398 |
| Qu JQ et al., 2009, China | n=300(150/150) | 7 | 50 m sprint | BF% | boy + BF%: *r* = 0.316**; girl + BF%: *r* = 0.169 |
| Raudsepp L et al., 1997, Estonia | n=215(0/215) | n_1_=7.3 ± 0.2  n_2_=8.5 ± 0.3  n_3_=9.4 ± 0.4  n_4_=10.2 ± 0.3 | 5×10 m SSR | SSF | 7y + SSF: *r* = 0.38**; 8y + SSF: *r* = 0.48**; 9y + SSF: *r* = 0.28*;  10y + SSF: *r* = 0.23 |
| Reigal RE et al., 2020a, Spain | n=167(81/86) | 14.53 ± 0.50 | 5×10 m SSR | Anxiety; Depression;  Physical self-concept | 5×10 m SSR + anxiety: *r* = 0.36***;  5×10 m SSR + depression: *r* = 0.06;  5×10 m SSR + physical self-concept: *r* = −0.38*** |

Table S2 (continued)

| Author(s), date, country | Sample(M/F) | Age (year) | Speed Test | Outcomes of interest | Findings |
| --- | --- | --- | --- | --- | --- |
| Reigal RE et al., 2020b, Spain | n=208(106/102) | 15.25 ± 0.74 | 5×10 m SSR | Anxiety; Depression | 5×10 m SSR + anxiety: *r* = 0.32**; 5×10 m SSR + depression: *r* = 0.08 |
| Reigal-Garrido RE et al., 2014, Spain | n=283(130/153) | 15.14 ± 0.76 | 50 m sprint | Physical self-concept | 50 m + physical self-concept: *r* = −0.55*** |
| Ruiz-Hermosa A et al., 2020, Spain | n=630(304/326) | 5.8 ± 0.4 | 4×10 m SSR | BMI | boy + BMI: *r* = 0.22**; girl + BMI: *r* = 0.23** |
| Szmodis M et al., 2019, Hungary | n_1_=1,116(1,116/0)  n_2_=1,643(1,643/0)  n_3_=1,496(1,496/0)  n_4_=1,429(1,429/0)  n_5_=1,235(1,235/0) | n_1_=9  n_2_=10  n_3_=11  n_4_=12  n_5_=13 | 30 m sprint | BF% | boy(9y) + BF%: *r* = 0.37***; boy(10y) + BF%: *r* = 0.42***;  boy(11y) + BF%: *r* = 0.42***; boy(12y) + BF%: *r* = 0.43***;  boy(13y) + BF%: *r* = 0.44*** |
| Tambalis K et al., 2013, Greek | n=141,169(72,086/69,083) | 7-10 | 30 m sprint | BMI | boy + BMI: *r* = 0.28***; girl + BMI: *r* = 0.23*** |
| Teng JL., 2019, China | n= 43,424(21,922/21,502) | 7-18 | 50 m sprint | BMI | boy + BMI: *r* = 0.212**; girl + BMI: *r* = 0.082* |
| Vandoni M et al., 2021, Italy | n=471(256/215) | 9.5 ± 1.12 | 20 m sprint | BMI  FM | 20 m + BMI: *r* = 0.1163; 20 m + FM: *r* = 0.0737 |
| Vanhelst J et al., 2016, France | n=1,851(946/905) | 12.3±3.6 | 50 m sprint | BMI | 50 m + BMI: *r* = 0.134*** |
| Vicente-Rodríguez G et al., 2008, Spain | n=278(109/169) | 13-18.5 | 4×10 m SSR | BMC | boy + BMC: *r* = -0.378**; girl + BMC: *r* = -0.275* |
| Xi W et al., 2013, China | n=8,098(4,068/4,030) | 7-18 | 50 m sprint | BMI | boy + BMI: *r* = 0.228**; girl + BMI: *r* = 0.087** |
| Wang H et al., 2018, China | n=2,116(1,261/855) | 3-6 | 2×10 m SSR | BMI | 2×10 m SSR + BMI: *r* = 0.203*** |
| Wang XL et al., 2018, China | n=43,650(21,822/21,828) | 7-18 | 50 m sprint | BMI | boy + BMI: *r* = 0.201**; girl + BMI: *r* = 0.080** |

Table S2 (continued)

| Author(s), date, country | Sample(M/F) | Age (year) | Speed Test | Outcomes of interest | Findings |
| --- | --- | --- | --- | --- | --- |
| Zaqout M et al., 2016, Europe | n=1635(822/813) | 8.4 ± 1.6 | 40 m sprint | WC; DBP; SBP  TG | 40 m + WC: *r* = 0.069**; 40 m + DBP: *r* = -0.003;  40 m + SBP: *r* = -0.071**; 40 m + TG: *r* = 0.102*** |
| Zhang Y et al., 2019, China | n_1_=107,206(56,749/50,457)  n_2_=70,213(37,343/32,870) | n_1_=10.38 ± 0.60  n_2_=14.46 ± 0.66 | 50 m sprint | BMI | n_1_+BMI: *r* = 0.19***; n_2_+BMI: *r* = 0.12*** |

*BMC* bone mineral content, *BMD* bone mineral density, *BMI* body mass index, *C-SoS* the speed of sound in the calcaneus, *DBP* diastolic blood pressure, *FFM* fat-free mass, *FM* fat mass, *M/F* male/female, *n* number of participants, *BF%* percentage of body fat, *R-SoS* the speed of sound in the third distal radius, *SBP* systolic blood pressure, *SSF* sum of skinfolds*, SSR* speed shuttle run, *TG* Triglyceride, *T-SoS* the speed of sound in the midshaft tibia, *WC* waist circumference, ** p*<0.05, ***p*<0.01, ****p*<0.001

# Table S3: The risk of bias assessment of the included studies

| Citation | Q1 | Q2 | Q3 | Q4 | Q5 | Q6 | Q7 | Q8 | Yes (%) | RoB |
| --- | --- | --- | --- | --- | --- | --- | --- | --- | --- | --- |
| Agha-Alinejad H et al., 2015 | x | ✓ | ✓ | ✓ | ✓ | ✓ | ✓ | ✓ | 87.5 | low |
| Andrade S et al., 2014 | ✓ | ✓ | ✓ | ✓ | x | x | ✓ | ✓ | 75 | low |
| Ara I et al., 2004 | x | ✓ | ✓ | ✓ | x | x | ✓ | ✓ | 62.5 | moderate |
| Arriscado D et al,2014 | x | ✓ | ✓ | ✓ | x | x | ✓ | ✓ | 62.5 | moderate |
| Baj-Korpak J et al., 2022 | x | ✓ | ✓ | ✓ | x | x | ✓ | ✓ | 62.5 | moderate |
| Batez M et al., 2021 | ✓ | ✓ | ✓ | ✓ | x | x | ✓ | ✓ | 75 | low |
| Becerra et al., 2013 | x | ✓ | ✓ | ✓ | x | x | ✓ | ✓ | 62.5 | moderate |
| Borrego-Balsalobre F J et al., 2022 | x | ✓ | ✓ | ✓ | x | x | ✓ | ✓ | 62.5 | moderate |
| Carraro A et al., 2010 | x | ✓ | ✓ | ✓ | x | x | ✓ | ✓ | 62.5 | moderate |
| Chen C et al., 1994 | x | ✓ | ✓ | ✓ | ✓ | ✓ | ✓ | ✓ | 87.5 | low |
| Colella D et al., 2009 | ✓ | ✓ | ✓ | ✓ | ✓ | ✓ | ✓ | ✓ | 100 | low |
| Du J et al., 2011 | x | ✓ | ✓ | ✓ | ✓ | ✓ | ✓ | ✓ | 87.5 | low |
| Esmaeilzadeh S et al., 2015 | ✓ | ✓ | ✓ | ✓ | x | x | ✓ | ✓ | 75 | low |
| García F G, Gómez M R et al., 2011 | x | ✓ | ✓ | ✓ | x | x | ✓ | ✓ | 62.5 | moderate |
| García-Sánchez A et al., 2013 | x | ✓ | ✓ | ✓ | ✓ | ✓ | ✓ | ✓ | 87.5 | low |
| Gonzalez-Suarez C B et al,2013 | ✓ | ✓ | ✓ | ✓ | ✓ | ✓ | ✓ | ✓ | 100 | low |
| Gómez-Bruton A et al., 2020 | ✓ | ✓ | ✓ | ✓ | x | x | ✓ | ✓ | 75 | low |
| Henriksson P et al., 2022 | ✓ | ✓ | ✓ | ✓ | x | x | ✓ | ✓ | 75 | low |
| Henriques-Neto D et al., 2020a | x | ✓ | ✓ | ✓ | ✓ | ✓ | ✓ | ✓ | 87.5 | low |
| Henriques-Neto D et al., 2020b | x | ✓ | ✓ | ✓ | ✓ | ✓ | ✓ | ✓ | 87.5 | low |

Table S3 (continued)

| Citation | Q1 | Q2 | Q3 | Q4 | Q5 | Q6 | Q7 | Q8 | Yes (%) | RoB |
| --- | --- | --- | --- | --- | --- | --- | --- | --- | --- | --- |
| Hensley LD et al., 1982 | x | ✓ | ✓ | ✓ | ✓ | ✓ | ✓ | ✓ | 87.5 | low |
| Hernández-Jaña S et al,2021 | ✓ | ✓ | ✓ | ✓ | x | x | ✓ | ✓ | 75 | low |
| Klein M et al., 2013 | x | ✓ | ✓ | ✓ | ✓ | ✓ | ✓ | ✓ | 87.5 | low |
| Kurtoğlu A et al., 2024 | ✓ | ✓ | ✓ | ✓ | x | x | ✓ | ✓ | 75 | low |
| Kwieciński J et al., 2018 | x | ✓ | ✓ | ✓ | ✓ | ✓ | ✓ | ✓ | 87.5 | low |
| Liu X H et al., 2018 | x | ✓ | ✓ | ✓ | x | x | ✓ | ✓ | 62.5 | moderate |
| Llagjeviq-Govori A et al., 2025 | x | ✓ | ✓ | ✓ | ✓ | ✓ | ✓ | ✓ | 87.5 | low |
| López Alonzo S J et al., 2022 | x | ✓ | ✓ | ✓ | x | x | ✓ | ✓ | 62.5 | moderate |
| López-Gil J F et al., 2020 | x | ✓ | ✓ | ✓ | ✓ | ✓ | ✓ | ✓ | 87.5 | low |
| Madi D M et al., 2018 | ✓ | ✓ | ✓ | ✓ | ✓ | ✓ | ✓ | ✓ | 100 | low |
| Malina RM et al., 2018 | x | ✓ | ✓ | ✓ | x | x | ✓ | ✓ | 62.5 | moderate |
| Martinez‐Tellez B et al., 2016 | ✓ | ✓ | ✓ | ✓ | x | x | ✓ | ✓ | 75 | low |
| Mello J B et al., 2022 | x | ✓ | ✓ | ✓ | x | x | ✓ | ✓ | 62.5 | moderate |
| Mello J B et al., 2023 | x | ✓ | ✓ | ✓ | x | x | ✓ | ✓ | 62.5 | moderate |
| Mendoza-Muñoz M et al., 2020 | ✓ | ✓ | ✓ | ✓ | x | x | ✓ | ✓ | 75 | low |
| Moliner-Urdiales D et al., 2011 | ✓ | ✓ | ✓ | ✓ | ✓ | ✓ | ✓ | ✓ | 100 | low |
| Moradi A et al., 2019 | ✓ | ✓ | ✓ | ✓ | x | x | ✓ | ✓ | 75 | low |
| Morano M et al., 2011 | x | ✓ | ✓ | ✓ | ✓ | ✓ | ✓ | ✓ | 87.5 | low |
| Moura‐Dos‐Santos M A et al., 2015 | x | ✓ | ✓ | ✓ | x | x | ✓ | ✓ | 62.5 | moderate |
| Muhumbe E et al., 2014 | ✓ | ✓ | ✓ | ✓ | ✓ | ✓ | ✓ | ✓ | 100 | low |

Table S3 (continued)

| Citation | Q1 | Q2 | Q3 | Q4 | Q5 | Q6 | Q7 | Q8 | Yes (%) | RoB |
| --- | --- | --- | --- | --- | --- | --- | --- | --- | --- | --- |
| Qiu S et al., 2024 | ✓ | ✓ | ✓ | ✓ | ✓ | ✓ | ✓ | ✓ | 100 | low |
| Qu J Q et al., 2009 | x | ✓ | ✓ | ✓ | ✓ | ✓ | ✓ | ✓ | 87.5 | low |
| Raudsepp L et al., 1997 | x | ✓ | ✓ | ✓ | ✓ | ✓ | ✓ | ✓ | 87.5 | low |
| Reigal R E et al., 2020a | ✓ | ✓ | ✓ | ✓ | x | x | ✓ | ✓ | 75 | low |
| Reigal R E et al., 2020b | ✓ | ✓ | ✓ | ✓ | x | x | ✓ | ✓ | 75 | low |
| Reigal-Garrido R E et al., 2014 | x | ✓ | ✓ | ✓ | x | x | ✓ | ✓ | 62.5 | moderate |
| Ruiz-Hermosa A et al., 2020 | x | ✓ | ✓ | ✓ | ✓ | ✓ | ✓ | ✓ | 87.5 | low |
| Szmodis M et al., 2019 | ✓ | ✓ | ✓ | ✓ | ✓ | ✓ | ✓ | ✓ | 87.5 | low |
| Tambalis K et al., 2013 | x | ✓ | ✓ | ✓ | ✓ | ✓ | ✓ | ✓ | 87.5 | low |
| Teng J L., 2019 | ✓ | ✓ | ✓ | ✓ | ✓ | ✓ | ✓ | ✓ | 100 | low |
| Vandoni M et al., 2021 | ✓ | ✓ | ✓ | ✓ | x | x | ✓ | ✓ | 75 | low |
| Vanhelst J et al., 2016 | x | ✓ | ✓ | ✓ | x | x | ✓ | ✓ | 62.5 | moderate |
| Vicente-Rodríguez G et al., 2008 | ✓ | ✓ | ✓ | ✓ | ✓ | ✓ | ✓ | ✓ | 100 | low |
| Xi W et al., 2013 | x | ✓ | ✓ | ✓ | x | x | ✓ | ✓ | 62.5 | moderate |
| Wang H et al., 2018 | x | ✓ | ✓ | ✓ | ✓ | ✓ | ✓ | ✓ | 87.5 | low |
| Wang X L et al., 2018 | ✓ | ✓ | ✓ | ✓ | ✓ | ✓ | ✓ | ✓ | 100 | low |
| Zaqout M et al., 2016 | ✓ | ✓ | ✓ | ✓ | ✓ | ✓ | ✓ | ✓ | 100 | low |
| Zhang Y et al., 2019 | x | ✓ | ✓ | ✓ | ✓ | ✓ | ✓ | ✓ | 87.5 | low |

x = “no”, ✓= “yes”

# Table S4: GRADE summary of findings

| outcome | n/*k* | Study design | Reasons for considering degrade certainty | | | | |  | Reasons for considering upgrade certainty | | | Certainty of evidence |
| --- | --- | --- | --- | --- | --- | --- | --- | --- | --- | --- | --- | --- |
|  |  |  | Risk of bias | Inconsistency | Indirectness | Imprecision | Publication bias |  | Large effect | Dose response | All plausible confounding and bias |  |
| **Anthropometric and adiposity parameters** | | | | | | | | | | | | |
| Body mass index | 30/57 | observational studies | No degrade | No degrade | No degrade | No degrade | No degrade |  | No upgrade | No upgrade | No upgrade | **⨁⨁◯◯** Low |
| Fat-free mass | 5/6 |  | No degrade | No degrade | Downgrade ^1^ | No degrade | No degrade |  | No upgrade | No upgrade | No upgrade | ⨁◯◯◯ Very low |
| Fat mass | 6/7 |  | No degrade | No degrade | No degrade | No degrade | No degrade |  | No upgrade | No upgrade | No upgrade | **⨁⨁◯◯** Low |
| Percentage of body fat | 11/24 |  | No degrade | No degrade | No degrade | No degrade | No degrade |  | No upgrade | No upgrade | No upgrade | **⨁⨁◯◯** Low |
| Sum of skinfolds | 5/20 |  | No degrade | No degrade | Downgrade ^2^ | No degrade | No degrade |  | No upgrade | No upgrade | No upgrade | ⨁◯◯◯ Very low |
| Waist circumference | 9/12 |  | No degrade | No degrade | No degrade | No degrade | No degrade |  | No upgrade | No upgrade | No upgrade | **⨁⨁◯◯** Low |
| **Cardiometabolic parameters** | | | | | | | | | | | | |
| Diastolic blood pressure | 3/3 | observational studies | No degrade | Downgrade ^3^ | Downgrade ^2^ | No degrade | No degrade |  | No upgrade | No upgrade | No upgrade | ⨁◯◯◯ Very low |
| Systolic blood pressure | 3/3 |  | No degrade | Downgrade ^3^ | Downgrade ^2^ | No degrade | No degrade |  | No upgrade | No upgrade | No upgrade | ⨁◯◯◯ Very low |
| Triglyceride | 3/3 |  | No degrade | No degrade | Downgrade ^2^ | No degrade | No degrade |  | No upgrade | No upgrade | No upgrade | ⨁◯◯◯ Very low |
| **Bone parameters** | | | | | | | | | | | | |
| Bone mineral content | 3/4 | observational studies | No degrade | No degrade | No degrade | No degrade | No degrade |  | No upgrade | No upgrade | No upgrade | ⨁⨁◯◯ Low |
| Bone mineral density | 3/3 |  | No degrade | No degrade | Downgrade ^4^ | No degrade | No degrade |  | No upgrade | No upgrade | No upgrade | ⨁◯◯◯ Very low |
| Bone speed of sound​ | 3/10 |  | No degrade | No degrade | Downgrade ^5^ | No degrade | No degrade |  | No upgrade | No upgrade | No upgrade | ⨁◯◯◯ Very low |
| **Psychological parameters** | | | | | | | | | | | | |
| Anxiety | 4/4 | observational studies | No degrade | No degrade | Downgrade ^6^ | No degrade | No degrade |  | No upgrade | No upgrade | No upgrade | ⨁◯◯◯ Very low |
| Depression | 4/4 |  | No degrade | Downgrade ^3^ | Downgrade ^6^ | No degrade | No degrade |  | No upgrade | No upgrade | No upgrade | ⨁◯◯◯ Very low |
| Physical self-concept | 4/4 |  | No degrade | No degrade | Downgrade ^6^ | No degrade | No degrade |  | No upgrade | No upgrade | No upgrade | ⨁◯◯◯ Very low |

*n* number of studies*, k* number of effect sizes

| 1. serious indirectness since more than 50% of population is from Asia | 1. serious indirectness since more than 50% of population is from Brazil. |
| --- | --- |
| 1. serious indirectness since more than 50% of population is from Europe | 1. serious indirectness since more than 50% of population is from Portugal. |
| 1. serious imprecision since 95% CI include the null value. | 1. serious indirectness since more than 50% of population is from Spain. |

# Figure S1: Forest plot evaluating the association between speed performance and body mass index

# Figure S2: Forest plot evaluating the association between speed performance and fat-free mass

# Figure S3: Forest plot evaluating the association between speed performance and fat mass

# Figure S4: Forest plot evaluating the association between speed performance and percentage of body fat

# Figure S5: Forest plot evaluating the association between speed performance and sum of skinfolds

# Figure S6: Forest plot evaluating the association between speed performance and waist circumference

# Figure S7: Forest plot evaluating the association between speed performance and diastolic blood pressure

# Figure S8: Forest plot evaluating the association between speed performance and systolic blood pressure

# Figure S9: Forest plot evaluating the association between speed performance and triglycerides

# Figure S10: Forest plot evaluating the association between speed performance and bone mineral content

# Figure S11: Forest plot evaluating the association between speed performance and bone mineral density

# Figure S12: Forest plot evaluating the association between speed performance and bone speed of sound​

# Figure S13: Forest plot evaluating the association between speed performance and anxiety

# Figure S14: Forest plot evaluating the association between speed performance and depression

# Figure S15: Forest plot evaluating the association between speed performance and physical self-concept

# Search strategy

Search equation: (child* OR youth* OR young* OR adolescen* OR teen* OR pediatric* OR preschool* OR student* OR pupil* OR boy* OR girl*) AND (speed OR "speed performance" OR "speed of movement" OR sprint OR dash OR "running speed" OR "speed agility" OR "speed-agility" OR "speed/agility" OR "speed shuttle run" OR "motor fitness" OR "skill-related fitness" OR "speed test") AND (health OR psych* OR benefit* OR risk OR consequence*) NOT (Athlete* OR player* OR older OR elder* OR disorder* OR disabilit* OR swim* OR walk* OR word OR read OR writ* OR Handwriting OR vehicle OR road OR driv* OR railway OR food OR drink OR diet OR noise OR army OR soldier OR animal* OR rat OR rats OR mouse OR mice OR rabbit*)

Restrictions: English, Chinese, or Spanish languages; published or accepted peer-reviewed journal articles; dates from inception to March 31, 2025.
